# Surface-Modified Cellulose Nanofibers for Stabilization of Barite in Water-Based Drilling Fluids Under High Temperatures

**DOI:** 10.3390/polym18192328

**Published:** 2026-09-24

**Authors:** Ziheng Zhang, Dongqing Yang, Ziyan Li, Yang Ding, Chaozheng Liu, Mei-Chun Li

**Affiliations:** 1 School of Petroleum Engineering, China University of Petroleum (East China), Qingdao 266580, China; 2 Co-Innovation Center of Efficient Processing and Utilization of Forest Resources, College of Materials Science and Engineering, Nanjing Forestry University, Nanjing 210037, China; 3 State Key Laboratory of Deep Oil and Gas, China University of Petroleum (East China), Qingdao 266580, China; 4 Shandong Key Laboratory of Oil and Gas Field Chemistry, China University of Petroleum (East China), Qingdao 266580, China

**Keywords:** drilling fluid, cellulose nanofibers, surface grafting, barite, high-temperature stability, biodegradability

## Abstract

Surface modification is an effective strategy for extending the application of cellulose nanofibers (CNFs) under harsh conditions while preserving their sustainable characteristics. Herein, thermally stable modified TEMPO-oxidized cellulose nanofibers (MTCNFs) were fabricated by free-radical graft copolymerization of 2-acrylamido-2-methylpropane sulfonic acid (AMPS) and acrylamide (AM) onto the CNF surface. Spectroscopic and microscopic characterizations confirmed the formation of a polymer-grafted shell enriched with sulfonic acid and amide functionalities. Surface grafting markedly enhanced the thermal stability of CNFs, while the zeta potential became more negative, indicating improved colloidal stability. The modified nanofibers effectively inhibited barite sedimentation after aging, as demonstrated by multiple light scattering and particle size analyses. Mechanistic investigations revealed that the superior suspension stability originated from the synergistic enhancement of electrostatic repulsion, steric hindrance provided by the grafted polymer chains, and the formation of a robust three-dimensional nanofiber network. When incorporated into water-based drilling fluids, MTCNFs significantly improved rheological stability, reduced filtration loss, and suppressed barite sagging under high-temperature conditions. Furthermore, the MTCNFs exhibited measurable biodegradation potential under the conditions employed. This work demonstrates that rational surface engineering of CNFs offers an effective route to simultaneously improve thermal stability and suspension performance, broadening the applicability of sustainable polysaccharide nanomaterials in high-temperature colloidal systems.

## 1. Introduction

The exploration of deep and ultra-deep oil and gas resources has led to increasingly demanding drilling environments characterized by elevated temperatures and pressures [1,2]. Bottomhole temperatures commonly exceed 150 °C and may surpass 180 °C in ultra-deep wells [3,4,5]. Under these harsh conditions, water-based drilling fluids (WBDFs) are required to simultaneously maintain rheological stability, filtration control, and homogeneous suspension of weighting materials to ensure wellbore pressure balance and drilling safety [6,7,8]. Barite is the most widely used weighting material because of its high density, low cost, and abundant availability [9]. However, prolonged exposure to high temperatures weakens the structural integrity of drilling fluids, promoting barite aggregation and sedimentation. The resulting density heterogeneity and rheological deterioration increase the risks of differential sticking, wellbore instability, and even well-control incidents [10,11,12]. Developing environmentally benign suspension stabilizers capable of maintaining colloidal stability under high-temperature conditions therefore remains a critical challenge for modern drilling fluids.

Currently, polymeric suspension stabilizers, e.g., xanthan gum [13] and partially hydrolyzed polyacrylamide (HPAM) [14,15], are commonly incorporated into WBDFs to retard barite settling by increasing the continuous-phase viscosity and constructing interconnected network structures. Nevertheless, most polymeric additives suffer from thermal degradation, hydrolysis, or chain scission under prolonged high-temperature exposure, resulting in rapid loss of rheological performance and suspension capability [16]. In addition, petroleum-derived polymers such as HPAM exhibit limited biodegradability and long environmental persistence, which conflicts with the growing demand for sustainable drilling-fluid technologies [17]. Consequently, renewable polysaccharide-based nanofibrils with superior suspending capacity and enhanced thermal stability have attracted increasing interest as next-generation drilling-fluid additives.

CNFs are renewable carbohydrate nanomaterials possessing high aspect ratios, abundant hydroxyl groups, large specific surface areas, and excellent mechanical properties [18,19,20,21]. These structural characteristics enable CNFs to establish percolated three-dimensional networks through hydrogen bonding and physical entanglement, thereby improving suspension stability and rheological behavior in aqueous systems. In recent years, CNFs have been extensively used as rheology modifiers, fluid-loss control agents, and suspension stabilizers in WBDFs [22,23,24,25,26,27]. Among various CNFs, TEMPO-oxidized cellulose nanofibers (T-CNFs) are particularly attractive because surface oxidation introduces abundant carboxylate groups that improve aqueous dispersibility, electrostatic stabilization, and adsorption onto bentonite particles [28,29]. The interconnected T-CNF network effectively suppresses barite particle sedimentation through a combination of electrostatic repulsion and network confinement [30]. However, the thermal stability of T-CNFs remains inadequate for deep-well applications. At elevated temperatures, decarboxylation and degradation of the oxidized cellulose backbone progressively weaken the hydrogen-bonded network, resulting in structural collapse and diminished suspension performance.

Surface functionalization offers an effective route to overcoming these limitations while preserving the intrinsic advantages of TCNFs. Among various modification strategies, graft copolymerization enables the introduction of functional polymer chains without disrupting the crystalline nanocellulose framework. 2-Acrylamido-2-methylpropane sulfonic acid (AMPS) is well known for its outstanding thermal stability, salt tolerance, and strongly dissociated sulfonic acid groups, which provide persistent surface charges and improved colloidal stability under harsh conditions [31]. Acrylamide (AM), in contrast, contributes abundant amide groups that enhance hydrogen bonding, hydration, and intermolecular interactions [32]. Synergistic grafting of AMPS and AM onto cellulose nanofibers is therefore expected to generate a thermally robust polymer shell capable of protecting the cellulose backbone while simultaneously strengthening electrostatic repulsion, steric stabilization, and hydration interactions [33]. Despite the widespread use of AMPS-based polymers in drilling fluids, systematic studies on AMPS/AM-grafted cellulose nanofibers and the relationship between their surface chemistry, thermal stability, and suspension behavior remain scarce.

In this work, AMPS and AM were grafted onto TCNFs through free-radical copolymerization to fabricate thermally stable modified cellulose nanofibers (MTCNFs). We hypothesize that the grafted AMPS/AM polymer shell not only protects the cellulose framework against thermally induced degradation but also creates a synergistic stabilization mechanism involving enhanced electrostatic repulsion, steric hindrance, and a reinforced three-dimensional nanofiber network. This synergistic effect is expected to preserve the colloidal stability of MTCNFs after prolonged exposure to high temperature and thereby significantly improve barite suspension, rheological stability, and filtration control in WBDFs. To test this hypothesis, the chemical structure, morphology, thermal stability, and surface charge of MTCNFs were systematically characterized. Its suspension performance was evaluated through multiple light scattering, particle-size analysis, and high-temperature aging tests, followed by drilling-fluid performance evaluation, mechanistic investigation, and biodegradability assessment. The findings establish a structure–property–performance relationship for surface-engineered cellulose nanofibers and provide a sustainable strategy for extending the application of carbohydrate nanomaterials to high-temperature colloidal systems.

## 2. Experimental Section

### 2.1. Materials

T-CNFs were supplied by Shengquan Group Co., Ltd. (Jinan, China). The nanofibers were produced from alkali-treated corn stover (cellulose purity >95 wt%) through TEMPO-mediated oxidation followed by mechanical fibrillation, during which the C6 primary hydroxyl groups (–CH_2_OH) were selectively converted into sodium carboxylate groups (–COO^−^). According to the supplier’s specifications, the individual nanofibrils in T-CNFs possessed widths ranging from 5 to 10 nm and lengths exceeding 10 μm. The nanofibers carried negatively charged sodium carboxylate groups with Na^+^ as the corresponding counter ions. The surface carboxylate density was approximately 0.60 mmol/g. AM, AMPS, ceric ammonium nitrate (CAN), and sodium hydroxide (NaOH) were purchased from Sinopharm Chemical Reagent Co., Ltd. (Shanghai, China) and used as received without further purification. Sodium bentonite (Na-Bent, API 13A grade) was used as the base clay for drilling fluid preparation. Potassium chloride (KCl), sodium carbonate (Na_2_CO_3_), calcium carbonate (CaCO_3_), and barite were of industrial grade. The commercial drilling-fluid additive DSP-1 was supplied by a local oilfield chemical manufacturer. Deionized water was used throughout all experiments. The schematic figure was prepared using Microsoft PowerPoint 2024 and Adobe Photoshop 2021.

### 2.2. Preparation of MTCNFs

MTCNFs were prepared through ceric ion-initiated free-radical graft copolymerization of AM and AMPS onto the surface of TCNFs. Briefly, 5.0 g of dried T-CNFs were dispersed in 150 mL of deionized water and stirred at 300 rpm for 30 min to obtain a homogeneous suspension. Subsequently, 1.5 g of CAN was introduced as the redox initiator and allowed to react with the hydroxyl groups on the cellulose surface under continuous stirring for 20 min. AM (2.10 g) and AMPS (4.14 g) were then sequentially added, and the suspension pH was adjusted to 8.0 using 1 mol/L NaOH solution. The graft copolymerization was conducted at 65 °C for 3 h under a nitrogen atmosphere. After completion of the reaction, the suspension was cooled to room temperature and centrifuged at 8000 rpm for 15 min. The resulting solid was washed three times with deionized water to remove unreacted monomers and soluble by-products, followed by one washing step with absolute ethanol. The purified product was vacuum-dried at 50 °C to constant weight, ground, and sieved to obtain the MTCNFs. A schematic illustration of the preparation procedure is presented in Figure 1.

### 2.3. Preparation of Barite Suspensions and WBDFs

To evaluate the suspension-stabilizing performance of cellulose nanofibers before and after surface modification, barite suspensions and WBDFs were prepared.

For the barite suspension, 100 g of barite was dispersed in 400 mL of deionized water, followed by the addition of 1 wt% T-CNFs or MTCNFs. The mixtures were stirred at 10,000 rpm for 20 min using a high-speed mixer until homogeneous suspensions were obtained.

The WBDFs were prepared by sequentially dispersing 3 wt% Na-Bent, 0.07 wt% Na_2_CO_3_, 5 wt% KCl, 5 wt% CaCO_3_, and 1 wt% DSP-1 in 400 mL of deionized water under continuous stirring at 10,000 rpm for 30 min. Barite was subsequently added to adjust the drilling-fluid density to 1.5 g/cm^3^. Finally, T-CNFs or MTCNFs (1 wt%) were incorporated into the formulated drilling fluid, and mixing was continued at 10,000 rpm for an additional 20 min until a uniform dispersion was achieved. The prepared drilling fluids were then subjected to subsequent rheological, filtration, suspension stability, and high-temperature aging evaluations.

### 2.4. Characterization of T-CNFs and MTCNFs

The chemical structures of T-CNFs and MTCNFs were characterized by Fourier-transform infrared spectroscopy (FT-IR, IRTRacer-100, SHIMADZU, Kyoto, Japan). Prior to analysis, the samples were vacuum-dried, finely ground, thoroughly mixed with KBr powder, and compressed into transparent pellets. FT-IR spectra were collected over the wavenumber range of 4000–400 cm^−1^.

The morphology of the T-CNFs and MTCNFs was examined using transmission electron microscopy (TEM, JEM-1400, JEOL Ltd., Tokyo, Japan). A dilute aqueous suspension (0.05 wt%) was ultrasonicated for 15 min and allowed to stand for 30 min to remove large aggregates. A drop of the supernatant was deposited onto an ultrathin carbon-coated copper grid and negatively stained with 2 wt% phosphotungstic acid for 2 min. The grid was subsequently rinsed with deionized water, air-dried, and observed by TEM.

The thermal stability of the T-CNFs and MTCNFs was evaluated by thermogravimetric analysis (TGA, TGA2, Mettler-Toledo GmbH, Greifensee, Switzerland) under a nitrogen atmosphere from 25 to 600 °C at a heating rate of 10 °C/min. In addition, MTCNFs recovered after dynamic aging at 180 °C were subjected to the same TGA procedure to evaluate the retention of thermal stability after high-temperature exposure.

The surface charge of the T-CNFs and MTCNFs was determined using a zeta potential analyzer (Zetasizer Nano Z, Malvern, UK). Measurements were performed on 0.01 wt% aqueous dispersions at 25 °C, and each value represents the average of three independent measurements.

### 2.5. Suspension Stabilization Performance of T-CNFs and MTCNFs

The prepared barite suspensions containing 1 wt% T-CNFs or MTCNFs were transferred into glass measuring cylinders. The cylinders were then placed either vertically or at a 45° inclination angle and allowed to stand undisturbed for 24 h. After the static period, fluid samples were carefully extracted from the top and bottom sections of each cylinder, and their densities were measured to calculate the sag factor (SF). For post-aging evaluations, the suspensions were dynamically aged in a roller oven at 180 °C for 16 h, cooled to room temperature, and subjected to the same static sag tests. The SF was calculated using Equation (1).

(1) $$SF=\frac{\rho_{bottom}}{\left(\rho_{bottom}+\rho_{top}\right)}$$

where $\rho_{bottom}$ is the density of the sample taken from the bottom of the cylinder (g·cm^−3^), $\rho_{top}$ is the density of the sample taken from the top of the cylinder (g·cm^−3^).

Dynamic light scattering (DLS) was employed to determine the hydrodynamic particle size and evaluate the influence of T-CNFs and MTCNFs on the dispersion state of barite particles.

The suspension stability of the barite systems in the presence of T-CNFs and MTCNFs was further investigated using a multiple light scattering (MLS) analyzer, which continuously monitored particle migration, aggregation, and sedimentation without disturbing the suspension, thereby enabling quantitative evaluation of colloidal stability. All static sag tests and suspension stability evaluations were performed in triplicate. The experimental data are presented as the mean values ± standard deviations to ensure reproducibility.

### 2.6. Performance of WBDFs Containing T-CNFs and MTCNFs

To simulate the high-temperature conditions encountered in deep and ultra-deep wells, the prepared WBDFs containing TCNFs and MTCNFs were dynamically aged in sealed stainless-steel aging cells at 180 °C using a roller oven. After aging, the samples were naturally cooled to room temperature before subsequent characterization. All subsequent performance characterizations, including suspension stability, rheological properties, and filtration tests, were conducted three times. The results are reported as the mean values ± standard deviations.

#### 2.6.1. Suspension Stability

The suspension stability of the drilling fluids was evaluated by measuring barite sag after static aging. The prepared fluids were transferred into graduated cylinders and allowed to stand undisturbed for 1 h. Samples were then collected from the top and bottom of the cylinders for density measurements. The SF was calculated using Equation (1), as well.

#### 2.6.2. Rheological Properties

The rheological properties of the drilling fluids were measured using a six-speed rotational viscometer (Model ZNN-D6, Qingdao Haitongda Special Instrument Co., Ltd., Qingdao, China) at room temperature. The dial readings were recorded for six rotational speeds of 600, 300, 200, 100, 6, and 3 rpm that corresponded to shear rates of 1022, 511, 341, 170.3, 10.22, and 5.11 s^−1^, respectively. All parameters were measured and calculated according to the American Petroleum Institute Recommended Practice API RP 13-1 (2009) [34]. The apparent viscosity (AV), plastic viscosity (PV), and yield point (YP) were described according to the Bingham fluid model as follows:

(2) $$AV=\frac{\theta_{600}}{2}$$

(3) $$PV=\theta_{600}-\theta_{300}$$

(4) $$YP=0.511(\theta_{300}-PV)$$

where AV is the apparent viscosity (mPa·s), PV is the plastic viscosity (mPa·s), YP is the yield point (Pa), and $\theta_{600}$ and $\theta_{300}$ are the viscometer dial readings at 600 rpm and 300 rpm, respectively. The gel strengths after 10 s and 10 min of static aging were determined from the 3 rpm dial readings.

#### 2.6.3. Filtration Properties

Filtration performance was evaluated using a standard API low-pressure filtration apparatus (Model SD-3, Qingdao Haitongda Special Instrument Co., Ltd., China) under a differential pressure of 100 psi for 30 min. The filtrate volume was recorded as the API fluid loss.

#### 2.6.4. Biodegradability

The environmental compatibility of the cellulose nanofibers and drilling-fluid systems was assessed using the BOD_5_/COD_Cr_ ratio. The biochemical oxygen demand (BOD_5_) measured after five days was determined using a respirometric BOD measuring system (Model OxiTop-i, WTW, Weilheim, Germany). The BOD_5_ tests were conducted at a constant incubation temperature of 20 °C. Prior to the measurements, the samples were inoculated with a microbial seed to initiate the biodegradation process. The chemical oxygen demand (COD_Cr_) was determined using the dichromate method with a COD reactor and a benchtop spectrophotometer (Models DRB200 and DR3900, Hach Company, Loveland, CO, USA).

## 3. Results and Discussion

### 3.1. Characterization of MTCNFs

#### 3.1.1. FTIR Analysis

FTIR spectroscopy was employed to investigate the chemical structure of T-CNFs before and after surface grafting and to verify the successful incorporation of AM and AMPS. As shown in Figure 2, both T-CNFs and MTCNFs exhibited the characteristic absorption bands of the cellulose backbone. The green shaded region (3000–3600 cm^−1^) highlights the broad stretching-vibration region associated mainly with O–H groups of cellulose and, after grafting, the overlapping N–H stretching vibrations introduced by AM and AMPS. The broad band centered at approximately 3448 cm^−1^ is assigned to the stretching vibration of hydroxyl groups (–OH), while the peak near 2900 cm^−1^ corresponds to the C–H stretching vibration of aliphatic groups [35,36,37]. The absorption band at 1612 cm^−1^ originates from the bending vibration of adsorbed water associated with the hydrophilic cellulose surface, whereas the band at 1062 cm^−1^ is attributed to the C–O–C stretching vibration of the β-(1→4)-glycosidic linkage and the C–O stretching vibration of hydroxyl groups [38].

Compared with T-CNF, MTCNF displayed several new absorption bands that provide clear evidence of successful surface functionalization. A distinct peak at approximately 1672 cm^−1^ is assigned to the stretching vibration of amide carbonyl groups (C = O), confirming the incorporation of polyacrylamide segments onto the cellulose surface [39]. In addition, the appearance of a band at 1041 cm^−1^, corresponding to the asymmetric stretching vibration of sulfonate groups (–SO_3_^−^), together with a weak absorption at 630 cm^−1^ attributed to C–S/S–O vibrations, demonstrates the successful introduction of AMPS-derived sulfonic acid functionalities [40]. These newly formed functional groups significantly increase the density of hydrophilic ionic and amide moieties on the nanofiber surface, which is expected to strengthen electrostatic repulsion and hydration interactions in aqueous media.

Importantly, no obvious changes were observed in the characteristic cellulose absorption bands after modification, suggesting that the free-radical graft copolymerization selectively introduced polymer chains onto the nanofiber surface while preserving the intrinsic cellulose framework. Such a surface-grafted architecture is advantageous because it combines the mechanical robustness of the cellulose core with the thermal stability and functional responsiveness of the AMPS/AM shell, thereby providing a structural basis for the enhanced colloidal stability and high-temperature suspension performance discussed in the following sections.

#### 3.1.2. Morphological Analysis

The morphology of T-CNFs and MTCNFs was examined by TEM, and the representative micrographs are presented in Figure 3a,b. As shown in Figure 3a, T-CNFs consist of well-individualized nanofibers with smooth surfaces and relatively uniform diameters. The nanofibers are highly interconnected through physical entanglement, forming a loose three-dimensional network in agreement with previous reports on TEMPO-oxidized CNFs [41]. Such a network is facilitated by the abundant surface carboxylate groups generated during TEMPO oxidation, which promote nanofiber dispersion through electrostatic repulsion while preserving extensive interfibrillar interactions [42].

After graft copolymerization with AM and AMPS, the overall fibrous morphology of cellulose was well retained, indicating that the modification process did not damage the intrinsic nanofiber framework (Figure 3b). In contrast to T-CNFs, MTCNFs exhibit a noticeably larger fiber diameter and a rougher surface texture. More importantly, a continuous low-contrast layer surrounding the cellulose core can be clearly distinguished, suggesting the formation of a polymer-grafted shell. This core–shell morphology provides direct morphological evidence that the graft copolymerization predominantly occurred on the nanofiber surface rather than within the cellulose crystalline domains, which is consistent with the FTIR results.

The formation of the polymer shell is expected to profoundly alter the interfacial properties of the cellulose nanofibers. The grafted AMPS/AM chains increase the effective hydrodynamic diameter of the nanofibers while introducing abundant sulfonate and amide groups that enhance hydration and interparticle interactions. Consequently, MTCNFs are expected to provide enhanced steric stabilization and hydration compared with pristine T-CNFs. These interfacial characteristics may contribute to reduced aggregation and improved dispersion. Combined with the post-aging thermal characterization and the suspension and rheological results obtained in the drilling-fluid systems, these observations support the proposed synergistic stabilization mechanism. Meanwhile, the preservation of the high-aspect-ratio cellulose core ensures the integrity of the interconnected nanofiber network, which is essential for stress transfer and suspension stabilization. The polymer shell can additionally serve as a protective interface that reduces thermally induced degradation of the cellulose surface, thereby improving the structural stability of the nanofiber network during high-temperature aging.

#### 3.1.3. Thermal Stability Analysis

The thermal stability of T-CNFs and MTCNFs was evaluated by TGA, and the corresponding results are presented in Figure 3c. Both samples exhibited the typical thermal degradation behavior of cellulose-based materials, involving an initial weight loss below approximately 120 °C associated with the evaporation of physically adsorbed water, followed by rapid decomposition of the cellulose framework at elevated temperatures. T-CNFs showed the onset of major thermal degradation at approximately 218 °C, which is characteristic of T-CNFs containing thermally labile carboxylate groups [43,44]. In contrast, MTCNFs exhibited a substantially higher onset decomposition temperature of 304 °C, representing an increase of 86 °C after surface grafting. Moreover, MTCNFs retained a higher residual mass throughout the principal degradation stage, particularly below 400 °C, indicating enhanced resistance to thermal decomposition [45]. To further evaluate whether this thermal protection was retained after exposure to the high-temperature aging condition, MTCNFs recovered after dynamic aging at 180 °C were additionally characterized by TGA. As shown in Figure 3c, the aged MTCNFs exhibited a decomposition temperature of approximately 281 °C. Although this value was lower than that of the unaged MTCNFs, it remained substantially higher than the major degradation temperature of T-CNFs.

The markedly improved thermal stability of MTCNFs is attributed to the formation of a polymer-grafted shell on the cellulose surface. The AMPS/AM chains introduce thermally stable sulfonate and amide functionalities that modify the interfacial environment of the nanofibers while preserving the cellulose backbone. This polymer layer is likely to restrict the mobility of cellulose chains and reinforce intermolecular interactions, thereby delaying the onset of thermal degradation [46]. In addition, the grafted shell may partially shield the oxidized cellulose surface from thermally induced degradation, contributing to the improved thermal resistance. The substantially increased decomposition temperature demonstrates that surface graft copolymerization effectively enhances the thermal stability of T-CNFs, which may contribute to maintaining the structural integrity of MTCNFs under high-temperature drilling-fluid conditions.

It should be noted that TGA measurements were conducted under a controlled nitrogen atmosphere and therefore do not directly reproduce the complex chemical environment of water-based drilling fluids, particularly the effects of dissolved salts, clay minerals, and other drilling-fluid additives. Accordingly, the TGA results are considered complementary evidence of the intrinsic thermal stability of MTCNFs rather than direct evidence of their complete stability under downhole conditions. The retention of functional performance after 180 °C aging was therefore further evaluated using rheological, suspension, and filtration measurements in the drilling-fluid system.

#### 3.1.4. Zeta Potential Analysis

The surface charge of T-CNFs and MTCNFs was evaluated by zeta potential measurements using dilute aqueous dispersions at 25 °C; the results are shown in Figure 3d. Compared with T-CNFs, MTCNFs exhibited a more negative zeta potential, indicating that surface grafting introduced additional ionizable groups and increased the electrostatic contribution to interparticle repulsion under the measurement conditions. The grafted AMPS chains are expected to contribute strongly dissociated sulfonate groups, while the AM component provides additional hydrophilic amide functionalities.

The enhanced surface charge can be attributed to the incorporation of AMPS-derived sulfonate groups, which possess stronger ionization ability than carboxyl groups and remain highly dissociated over a broad range of solution conditions [47]. As a result, MTCNF exhibits stronger electrostatic repulsion between adjacent nanofibers, thereby reducing the tendency for aggregation. Because colloidal dispersions with absolute zeta potential values greater than approximately 30 mV are generally considered electrostatically stable [48], both samples exhibited relatively high absolute zeta potential values under the measurement conditions, suggesting a substantial electrostatic contribution to colloidal stabilization. MTCNFs showed a more negative zeta potential, indicating enhanced electrostatic stabilization relative to T-CNFs under these dilute aqueous conditions. However, the zeta potential measurements were performed in dilute aqueous dispersions at room temperature and therefore do not directly reproduce the high-temperature and high-ionic-strength environment of WBDFs. The measured zeta potential should thus be regarded as evidence for the intrinsic interfacial charge characteristics of MTCNFs rather than a direct quantitative measure of their colloidal stability under downhole conditions. The latter was evaluated more directly through DLS/MLS measurements and the suspension performance of barite-containing systems before and after thermal aging.

### 3.2. Suspension Stabilization Performance of MTCNFs

#### 3.2.1. Evaluation of Suspension Stability

To evaluate the effect of surface grafting on the suspension-stabilizing capability of cellulose nanofibers, the sag factor (SF) of barite suspensions containing T-CNFs or MTCNFs was systematically determined under both vertical and 45° inclined conditions. As shown in Figure 4a,b, both nanofibers improved the suspension stability of the barite system; however, MTCNFs consistently exhibited lower SF values than T-CNFs, indicating a more homogeneous particle distribution and enhanced resistance to sedimentation. Under vertical static conditions, the SF decreased from 0.510 for the T-CNF suspension to 0.504 after surface grafting. A similar trend was observed under the 45° inclined condition, where the MTCNF suspension exhibited an SF of 0.508, compared with 0.515 for T-CNF. More importantly, after dynamic aging at 180 °C, the MTCNF suspension maintained low SF values of 0.508 and 0.51, whereas the SF values of the T-CNF suspension increased to 0.523 and 0.528, indicating that the grafted nanofibers effectively retained their suspension capability under severe thermal conditions.

The excellent high-temperature suspension performance of MTCNFs compares favorably with previously reported bio-based suspension stabilizers. Xu et al. reported that conventional cellulose- and starch-based additives undergo pronounced thermal degradation at 180 °C, resulting in the collapse of their colloidal network and loss of suspension capability [49]. Similarly, Mohamed et al. achieved an SF of approximately 0.50 using a urea-based anti-settling additive; however, the evaluation was limited to 121 °C [50]. In contrast, MTCNFs maintained comparable suspension stability after aging at 180 °C, demonstrating that surface grafting substantially extends the thermal operating window of T-CNFs for high-temperature applications.

The improved suspension stability originates from the synergistic modification of the nanofiber interface achieved through AMPS/AM grafting. TEMPO oxidation introduces abundant carboxyl groups that serve as reactive sites for surface graft copolymerization [51] while the grafted polymer chains generate a hydrated shell enriched with sulfonate and amide functionalities. This surface architecture enhances colloidal stability through multiple complementary mechanisms. First, the increased density of ionized sulfonate groups strengthens electrostatic repulsion between adjacent nanofibers and between nanofibers and barite particles, thereby suppressing particle aggregation. Second, the grafted polymer chains create a steric barrier that reduces direct particle-particle contact and inhibits thermally induced flocculation. Finally, the improved thermal stability of the modified nanofibers enables the interconnected cellulose network to remain intact during high-temperature aging, providing persistent mechanical support that effectively restrains gravitational settling of barite. In contrast, although T-CNFs possess a relatively high surface charge, the absence of a protective polymer shell makes its network more susceptible to thermal degradation, resulting in weakened structural integrity and diminished suspension performance after aging [52].

#### 3.2.2. Particle Size Distribution Analysis

The influence of surface modification of T-CNFs on barite particle dispersion was further investigated by dynamic light scattering, and the particle-size distributions after thermal aging are shown in Figure 5. The T-CNF/barite suspension exhibited a broad distribution with a characteristic peak centered at 1752 nm, suggesting the presence of relatively large particle aggregates. By comparison, the MTCNF/barite suspension displayed a narrower distribution with a dominant peak at 1570 nm and a substantially higher peak intensity, indicating a more homogeneous particle population.

The reduction in aggregate size and narrowing of the particle-size distribution demonstrate that graft modification effectively suppresses thermally induced flocculation of barite particles. The AMPS-derived sulfonate groups enhance electrostatic stabilization, while the grafted polymer chains provide steric repulsion that minimizes direct particle-particle contact [53]. At the same time, the improved thermal stability of MTCNF preserves the integrity of the nanofiber network during aging, enabling the modified cellulose to maintain uniform particle dispersion even under harsh thermal conditions [54]. These results are consistent with the zeta potential analysis and further confirm that surface grafting substantially improves the colloidal stability of T-CNF suspensions.

#### 3.2.3. Multiple Light Scattering Analysis

The sedimentation behavior of the aged suspensions was further evaluated by multiple light scattering (MLS). As shown in Figure 6a, the T-CNF/barite suspension exhibited pronounced variations in light transmittance along the sample height, indicating progressive particle migration and sedimentation after thermal aging. The large transmittance gradient reflects the formation of concentration differences within the suspension, suggesting that the original nanofiber network was unable to maintain colloidal stability under high-temperature conditions.

In contrast, the MTCNF/barite suspension (Figure 6b) displayed an almost constant transmittance profile throughout the sample height, indicating negligible particle migration during the measurement period. The uniform optical response confirms that the modified nanofibers effectively stabilized the barite particles against sedimentation. This enhanced stability can be attributed to the synergistic action of the polymer-grafted shell, which increases electrostatic repulsion and steric stabilization while preserving the interconnected nanofiber network after thermal aging. Consequently, MTCNF maintains a homogeneous colloidal structure and effectively suppresses gravitational settling under harsh conditions.

The overall stability of the suspensions was quantitatively assessed using the Turbiscan Stability Index (TSI), and the results are presented in Figure 7. A lower TSI value indicates greater colloidal stability. The T-CNF/barite suspension exhibited a continuous increase in TSI during the monitoring period, demonstrating progressive destabilization caused by particle aggregation and sedimentation after thermal aging. In contrast, the MTCNF/barite suspension maintained the lowest TSI values with only minimal variation throughout the test, indicating excellent resistance to structural destabilization.

#### 3.2.4. Proposed Stabilization Mechanisms

Based on the structural characterization and suspension performance results, a mechanism is proposed to explain the enhanced high-temperature suspension stability of the MTCNF/barite system (Figure 8). In the unmodified T-CNF suspension, elevated temperatures accelerate Brownian motion and increase the frequency of particle collisions, thereby promoting barite aggregation and sedimentation. At the same time, thermal degradation of the oxidized cellulose gradually weakens the hydrogen-bonded nanofiber network, resulting in reduced structural integrity and diminished capability to immobilize suspended particles. Consequently, the combined effects of particle aggregation and network disruption lead to progressive deterioration of the suspension stability after thermal aging. In contrast, surface grafting with AMPS and AM fundamentally modifies the interfacial properties of cellulose nanofibers, enabling MTCNF to stabilize barite suspensions through the synergistic contribution of electrostatic repulsion, steric stabilization, and a thermally robust nanofiber network.

(1) Enhanced electrostatic stabilization: The grafted AMPS chains introduce abundant sulfonate groups onto the cellulose surface. Compared with the carboxylate groups of T-CNFs, sulfonate groups possess stronger ionization ability and retain a high degree of dissociation under harsh conditions, resulting in a more negative surface potential. The increased surface charge strengthens electrostatic repulsion between adjacent nanofibers and between nanofibers and barite particles, thereby reducing thermally induced aggregation and maintaining a more homogeneous particle distribution [55].
(2) Steric stabilization through a hydrated polymer shell: Graft copolymerization forms a polymer shell enriched with amide and sulfonate functionalities on the nanofiber surface. These hydrophilic groups strongly interact with surrounding water molecules to establish a stable hydration layer, while the grafted polymer chains generate steric hindrance that prevents close particle–particle contact. The combined hydration and steric effects effectively suppress secondary aggregation of both nanofibers and barite particles, preserving colloidal stability even after prolonged exposure to elevated temperatures [56].
(3) Preservation of the three-dimensional nanofiber network: The improved thermal stability of MTCNF enables the interconnected cellulose network to retain its structural integrity during high-temperature aging. The grafted polymer chains increase inter-fiber entanglement and provide additional hydrogen-bonding sites through the amide groups, reinforcing the physical network formed by the cellulose nanofibers. This robust network provides continuous mechanical support for suspended barite particles while restricting their long-range migration under gravity. Rather than acting solely through viscosity enhancement, the interconnected nanofiber framework stabilizes the suspension by creating a spatial confinement network that uniformly distributes particles throughout the continuous phase, thereby minimizing sedimentation [50].

Collectively, these three mechanisms operate cooperatively to establish a highly stable colloidal system. The enhanced electrostatic repulsion minimizes particle aggregation, the hydrated polymer shell provides persistent steric stabilization, and the reinforced nanofiber network supplies long-term structural support. This structure–property–performance relationship highlights the potential of MTCNFs for high-temperature drilling fluid applications.

### 3.3. Performance of WBDFs Containing MTCNFs

To evaluate whether the improved physicochemical properties of MTCNFs could be translated into practical performance, the MTCNFs were incorporated into a model WBDF, which consists of 400 mL deionized water + 3 wt% Na-Bent + 0.07 wt% Na_2_CO_3_ + 5 wt% KCl + 5 wt% CaCO_3_ + 1 wt% DSP-1 polymer treatment agent, with barite added to achieve a density of 1.5 g/cm^3^. Three formulations were investigated: the base WBDF, WBDF containing 1 wt% T-CNFs, and WBDF containing 1 wt% MTCNFs. The rheological properties, filtration performance, and suspension stability of the three systems were systematically evaluated before and after thermal aging at 180 °C.

#### 3.3.1. Suspension Stability

The suspension stability of the drilling fluids was evaluated using the sag factor, and the results are shown in Figure 9a. Before thermal aging, the addition of 1 wt% T-CNFs reduced the sag factor of the base WBDF from 0.526 to 0.511, whereas MTCNFs further decreased the value to 0.509, indicating improved suspension of barite particles. After aging at 180 °C, the base WBDF exhibited pronounced sedimentation, with the sag factor increasing to 0.541. The WBDF containing T-CNFs also showed a noticeable increase in sag factor to 0.527, suggesting that the suspension network was partially disrupted during thermal aging. In contrast, the MTCNF-containing system maintained a substantially lower sag factor of 0.515, demonstrating superior resistance to thermally induced particle settling.

The improved suspension stability of the MTCNF-containing drilling fluid is consistent with the stabilization mechanism established for the model barite suspensions. The grafted AMPS/AM chains are expected to contribute to electrostatic repulsion and steric stabilization, while the thermally reinforced nanofiber network may provide additional physical confinement of suspended particles. The improved sag stability, rheological retention, and filtration performance after 180 °C aging are consistent with this proposed synergistic mechanism.

#### 3.3.2. Rheological Properties

The rheological properties of the drilling fluids are summarized in Figure 9b–e. Before thermal aging, the incorporation of both T-CNFs and MTCNFs increased the apparent viscosity (AV), plastic viscosity (PV), and yield point (YP) of the base fluid, demonstrating the ability of cellulose nanofibers to reinforce the drilling-fluid microstructure. Owing to their high aspect ratio and abundant surface functional groups, the nanofibers readily formed an interconnected three-dimensional network through physical entanglement and hydrogen bonding, thereby enhancing the load-bearing capability of the suspension.

After dynamic aging at 180 °C, the rheological properties of the base WBDF and the T-CNF-containing system deteriorated markedly. The YP of the WBDF/T-CNF system decreased from 14 Pa to 1.5 Pa, accompanied by a sharp reduction in the YP/PV ratio to 0.09, indicating a substantial loss of structural integrity and suspension capacity. This deterioration is attributed to the limited thermal stability of T-CNFs, whose oxidized surface functionalities and hydrogen-bonded network become progressively destabilized under prolonged thermal exposure. In contrast, the WBDF containing MTCNFs retained substantially higher rheological parameters after aging, maintaining an AV of 30 mPa·s, a YP of 8.176 Pa, and a YP/PV ratio of 0.372. These results demonstrate that surface grafting effectively improves the thermal durability of cellulose nanofibers and enables them to preserve the interconnected network responsible for rheological enhancement under harsh conditions.

#### 3.3.3. Filtration Performance

The filtration performance of the WBDFs before and after thermal aging is presented in Figure 9f. Before aging, all formulations exhibited comparable API filtration volumes ranging from 24 to 28 mL, indicating that the incorporation of cellulose nanofibers had only a limited influence on filtration under ambient conditions. After dynamic aging at 180 °C, however, the filtration behavior differed markedly among the three systems. The base WBDF exhibited a substantial increase in filtration volume to 60 mL, reflecting severe deterioration of the filter-cake structure after thermal exposure. In contrast, the WBDFs containing MTCNFs exhibited a remarkably low filtration volume of 12 mL, demonstrating excellent filtration control under high-temperature conditions.

The superior filtration performance of MTCNFs is attributed to its surface-engineered nanofiber architecture. The high-aspect-ratio cellulose framework promotes the formation of an interconnected network within the filter cake, while the grafted AMPS/AM polymer shell enhances hydration and interfacial interactions between the nanofibers and solid particles. These combined effects facilitate the formation of a more compact and less permeable filter cake, effectively reducing pore connectivity and restricting filtrate transport. Moreover, the improved thermal stability of MTCNFs enables the nanofiber network to retain its structural integrity during high-temperature aging, thereby preserving its sealing capability. These observations indicate that surface grafting enhances not only the colloidal stability of cellulose nanofibers but also their effectiveness as sustainable filtration-control additives under harsh conditions.

### 3.4. Biodegradability Assessment

The biodegradability of the cellulose nanofibers and the corresponding WBDF systems was evaluated using the BOD_5_/COD_Cr_ ratio, and the results are summarized in Table 1. According to the biodegradability classification adopted from previous drilling-fluid studies [57]. BOD_5_/COD_Cr_ ratios below 5%, 5–15%, 15–25%, and above 25% correspond to low, relatively low, degradable, and easily degradable categories, respectively. The BOD_5_/COD_Cr_ ratios of T-CNFs and MTCNFs were 47.5% and 45.1%, respectively, whereas the corresponding values for the WBDF/T-CNF and WBDF/MTCNF systems were 26.9% and 20.1%, respectively. These results indicate that the tested cellulose-based materials and drilling-fluid formulations exhibited measurable biodegradation potential under the conditions employed in the BOD_5_/COD_Cr_ assessment. These results demonstrate that surface grafting with AMPS and AM does not substantially compromise the environmental compatibility of cellulose nanofibers.

The favorable biodegradability of both materials originates from the intrinsic polysaccharide backbone of cellulose. Cellulose consists of β-(1→4)-linked glucan chains that can be efficiently hydrolyzed by cellulolytic microorganisms in natural environments [58]. Although surface grafting with AMPS and AM introduces synthetic polymer components, the characteristic cellulose framework is retained, as supported by the FTIR results, and may therefore contribute to the measurable biodegradation potential of MTCNFs. In the isolated-material test, MTCNFs exhibited a BOD_5_/COD_Cr_ ratio of 45.1%, whereas this value decreased to 20.1% after incorporation into the more chemically complex WBDF formulation, indicating that the presence of other drilling-fluid components affected the measured biodegradation response. The hydrophilic functional groups introduced by AMPS and AM may also influence water accessibility and interactions with microorganisms; however, the contribution of the grafted polymer chains to the overall biodegradation behavior cannot be quantitatively distinguished from that of the cellulose backbone based on the present BOD_5_/COD_Cr_ measurements alone. In contrast, conventional partially hydrolyzed polyacrylamide (HPAM) contains a carbon–carbon backbone that is considerably more resistant to microbial degradation, resulting in greater environmental persistence [59,60]. It should therefore be emphasized that the BOD_5_/COD_Cr_ ratio reflects biodegradation behavior under the specific test conditions and should not be interpreted as direct evidence of complete environmental degradation in natural or field environments. Overall, the present results support the measurable biodegradation potential and environmental compatibility of the investigated cellulose-based materials and drilling-fluid formulations, while further standardized long-term degradation and ecotoxicological studies are required to fully establish their environmental fate.

## 4. Conclusions

In this study, MTCNFs were successfully prepared by grafting AMPS and AM onto T-CNFs through free-radical graft copolymerization. Structural characterization confirmed the formation of a polymer-grafted core–shell architecture while preserving the cellulose nanofiber framework. Surface grafting significantly enhanced the physicochemical properties of the nanofibers, increasing the onset thermal decomposition temperature from 218 to 304 °C and shifting the zeta potential from −31.6 to −38.2 mV, indicating improved thermal stability and colloidal stability. The modified nanofibers exhibited markedly enhanced suspension stabilization after thermal aging at 180 °C. Compared with T-CNFs, MTCNFs more effectively suppressed barite aggregation and sedimentation, maintaining a sag factor of 0.508 after aging. Comprehensive characterization and suspension analyses indicate that the improved performance originates from the synergistic effects of enhanced electrostatic repulsion, hydrated steric stabilization, and preservation of an interconnected three-dimensional nanofiber network under high-temperature conditions. When incorporated into WBDFs, MTCNFs effectively improved rheological stability, reduced filtration loss to 12 mL, and maintained a yield point of 8.176 Pa and a sag factor of 0.515 after thermal aging, demonstrating that the enhanced colloidal stability of the modified nanofibers can be translated into improved macroscopic fluid performance. Furthermore, the BOD_5_/COD_Cr_ ratio of the MTCNF-containing drilling fluid remained 20.1%, indicating that surface grafting preserved the degradable nature of the cellulose-based material.

Overall, this work demonstrates that surface graft engineering is an effective strategy for simultaneously improving the thermal stability, colloidal stability, and functional performance of cellulose nanofibers while maintaining their environmental compatibility. The proposed modification approach broadens the application potential of sustainable carbohydrate nanomaterials in high-temperature colloidal systems and provides a promising route for developing bio-based functional additives for demanding industrial environments, including deep-well drilling and geothermal energy applications.

## Figures and Tables

**Figure 1:**
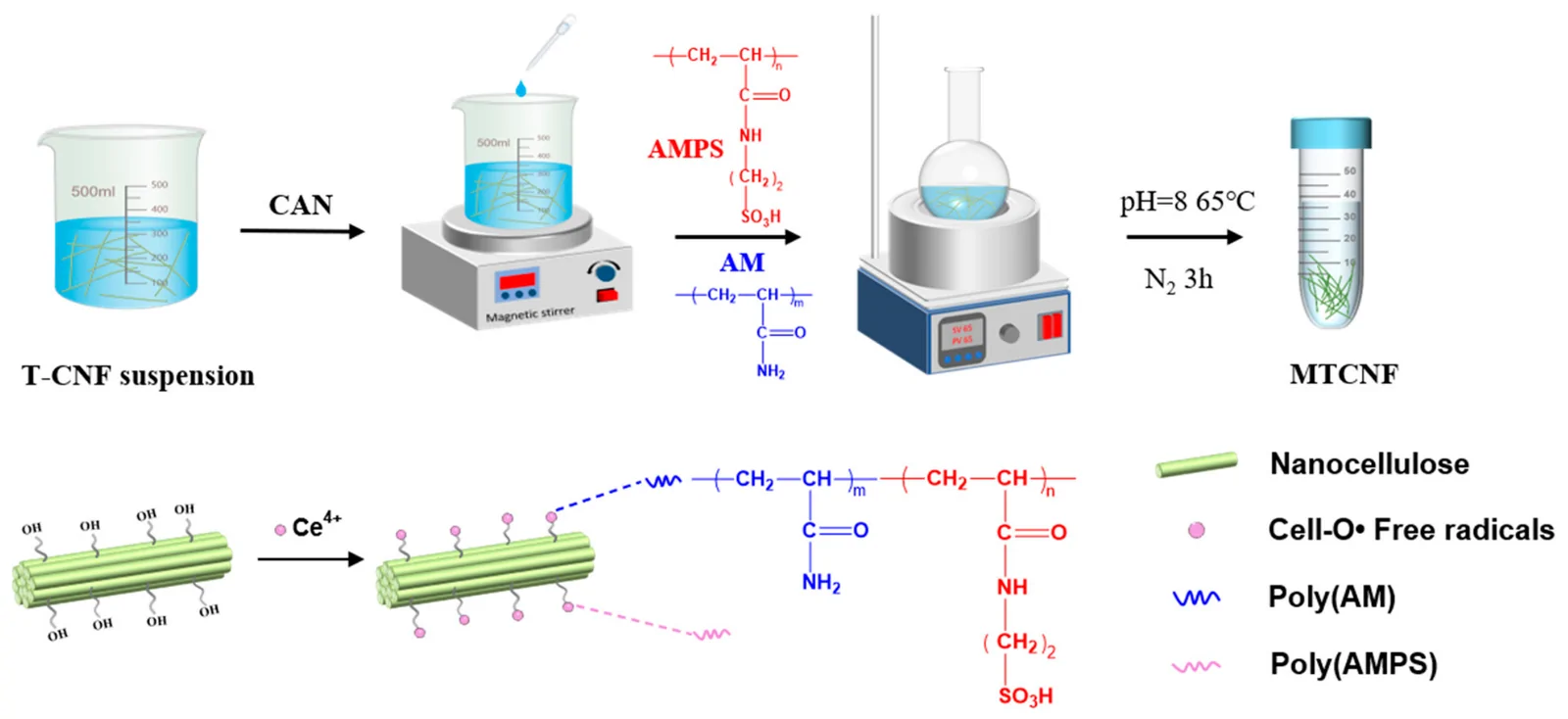
Schematic illustration of the synthesis of MTCNFs.

**Figure 2:**
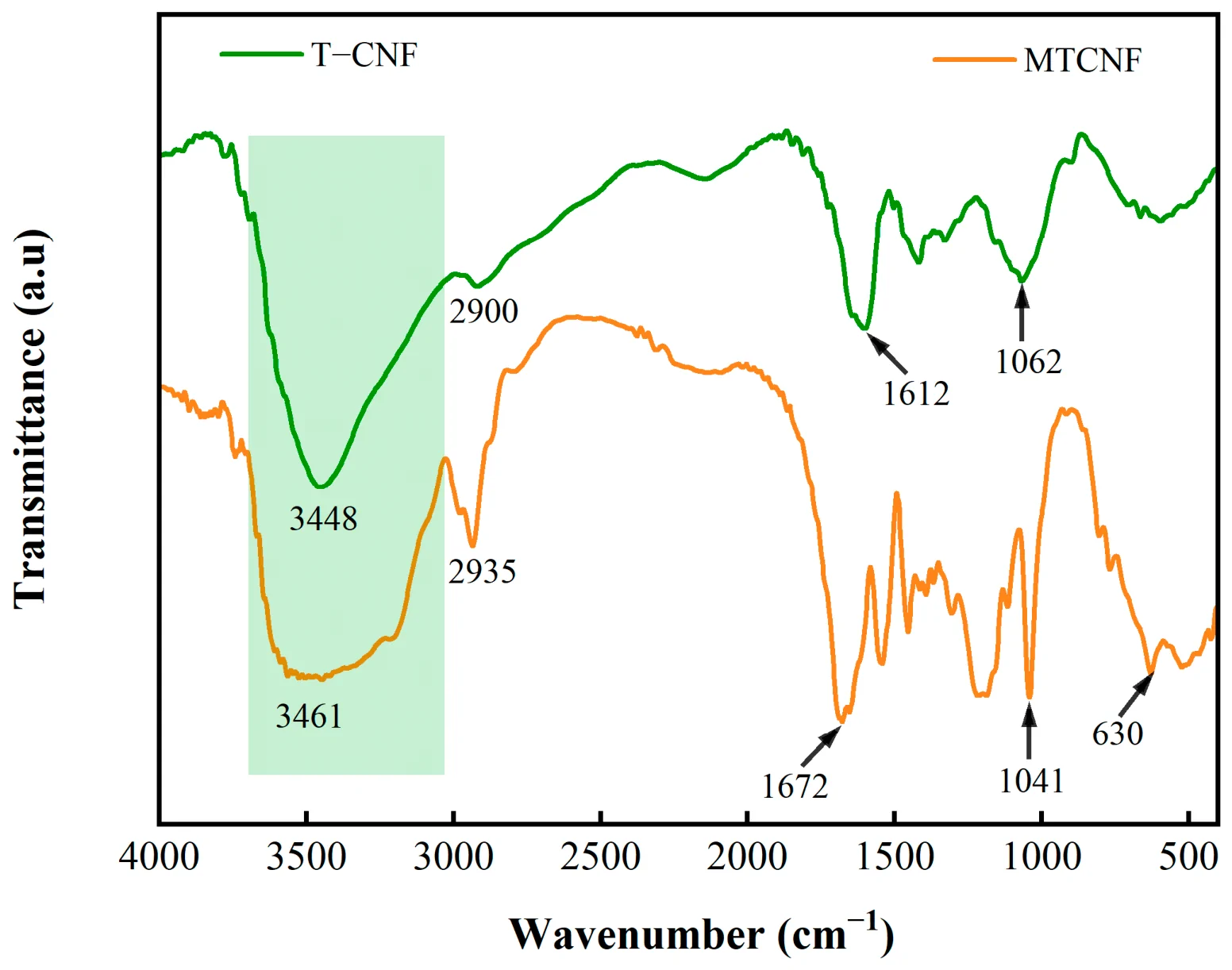
FTIR spectra of T-CNFs and MTCNFs.

**Figure 3:**
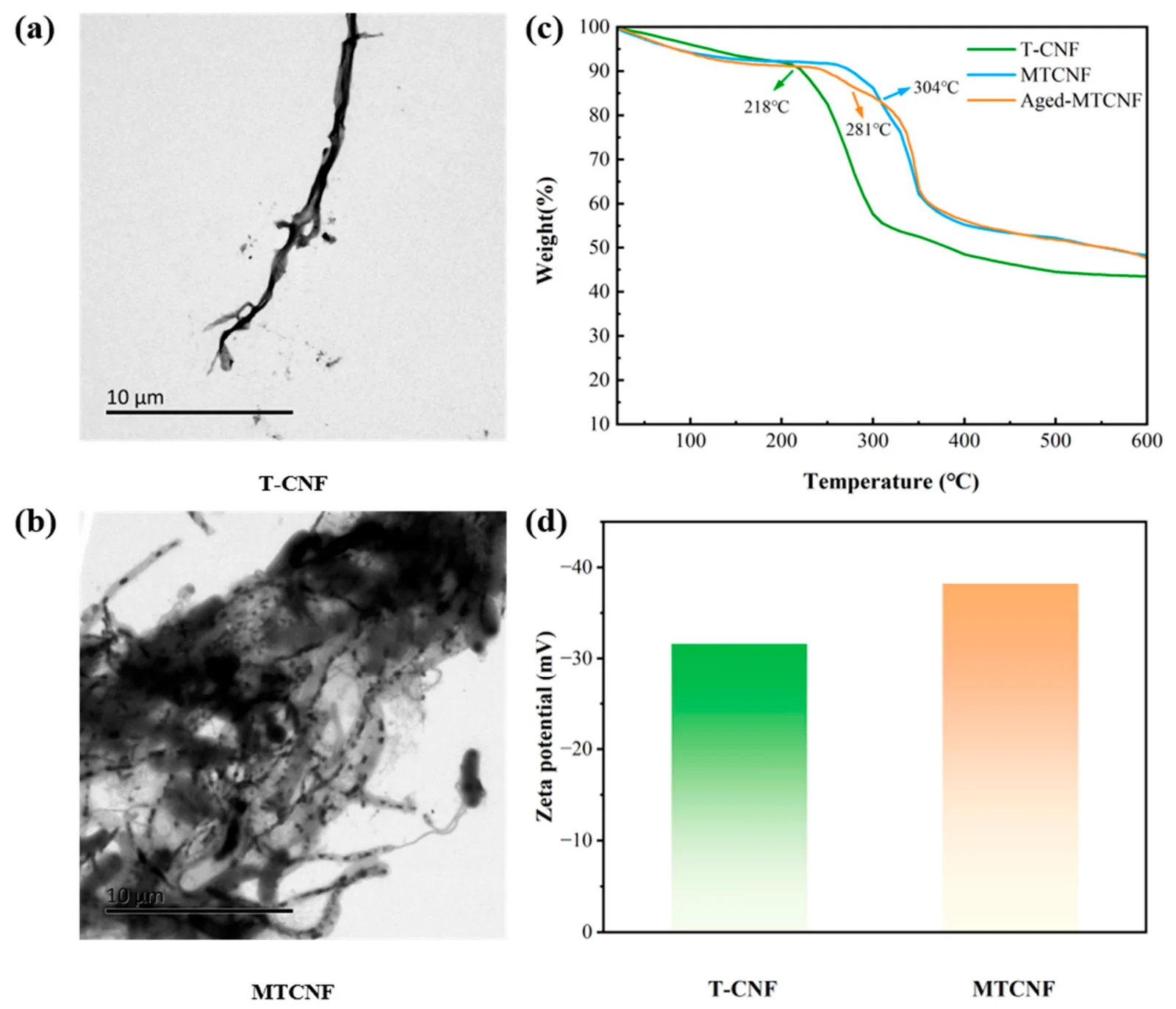
Morphology and physicochemical properties of T-CNFs and MTCNFs: (**a**) TEM micrograph of T-CNFs, (**b**) TEM micrograph of MTCNFs, (**c**) TGA curves, (**d**) zeta potential values.

**Figure 4:**
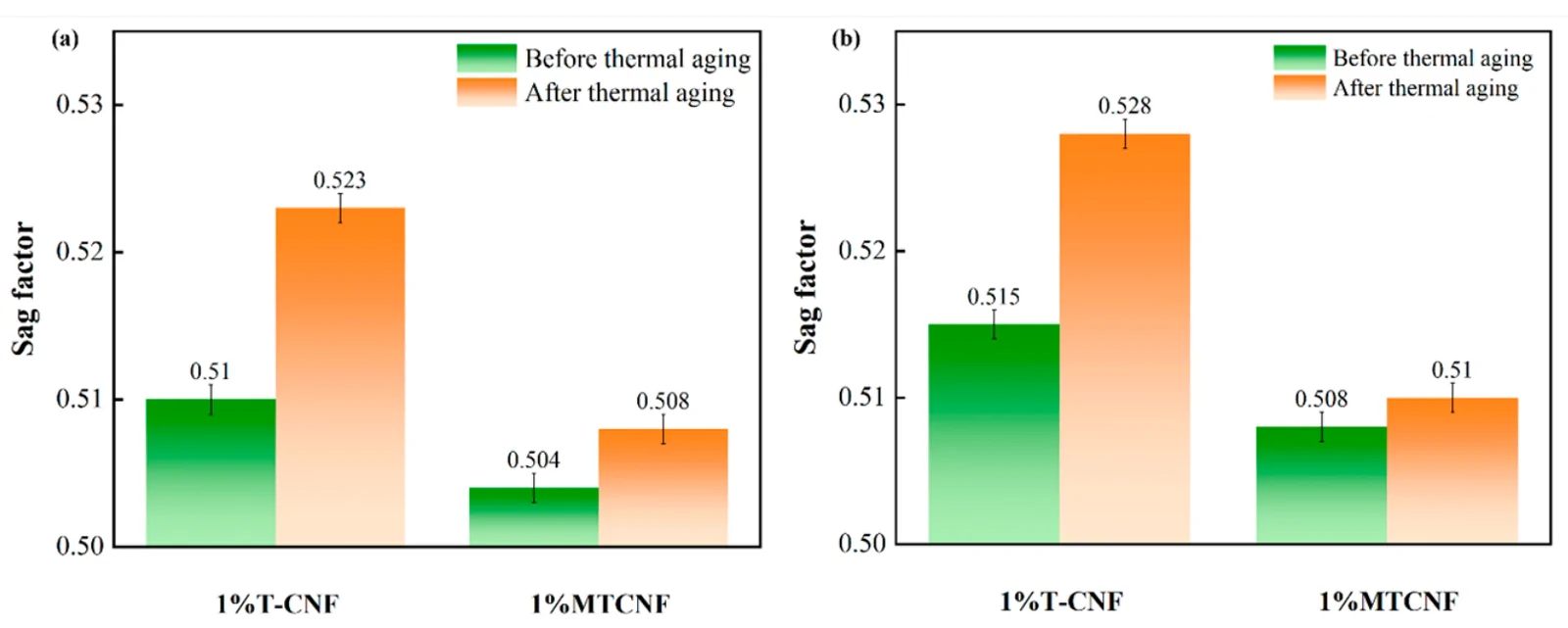
Sag factors before and after thermal aging under vertical (**a**) and 45° inclined (**b**) conditions.

**Figure 5:**
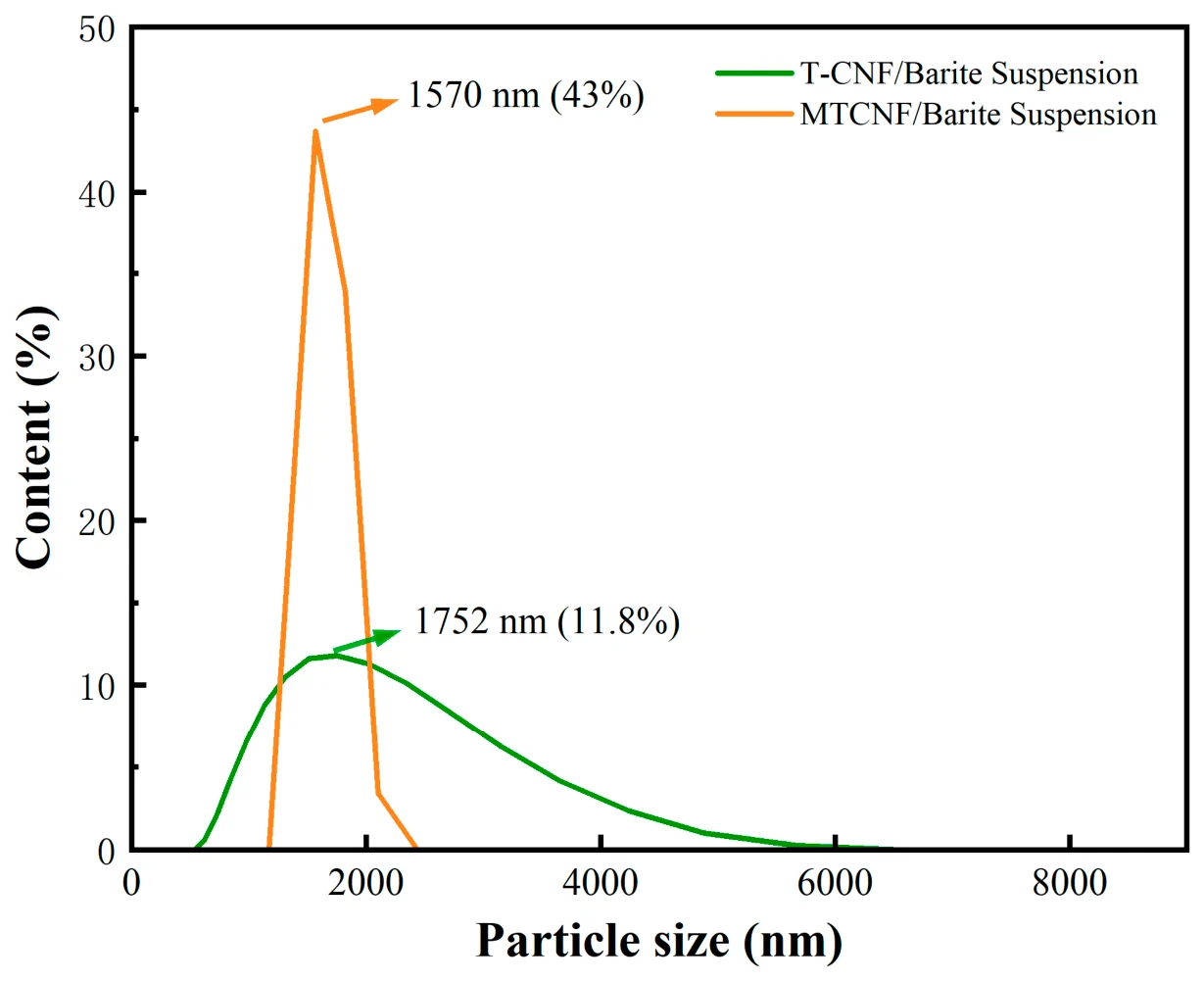
Particle size distributions (PSD) after thermal aging.

**Figure 6:**
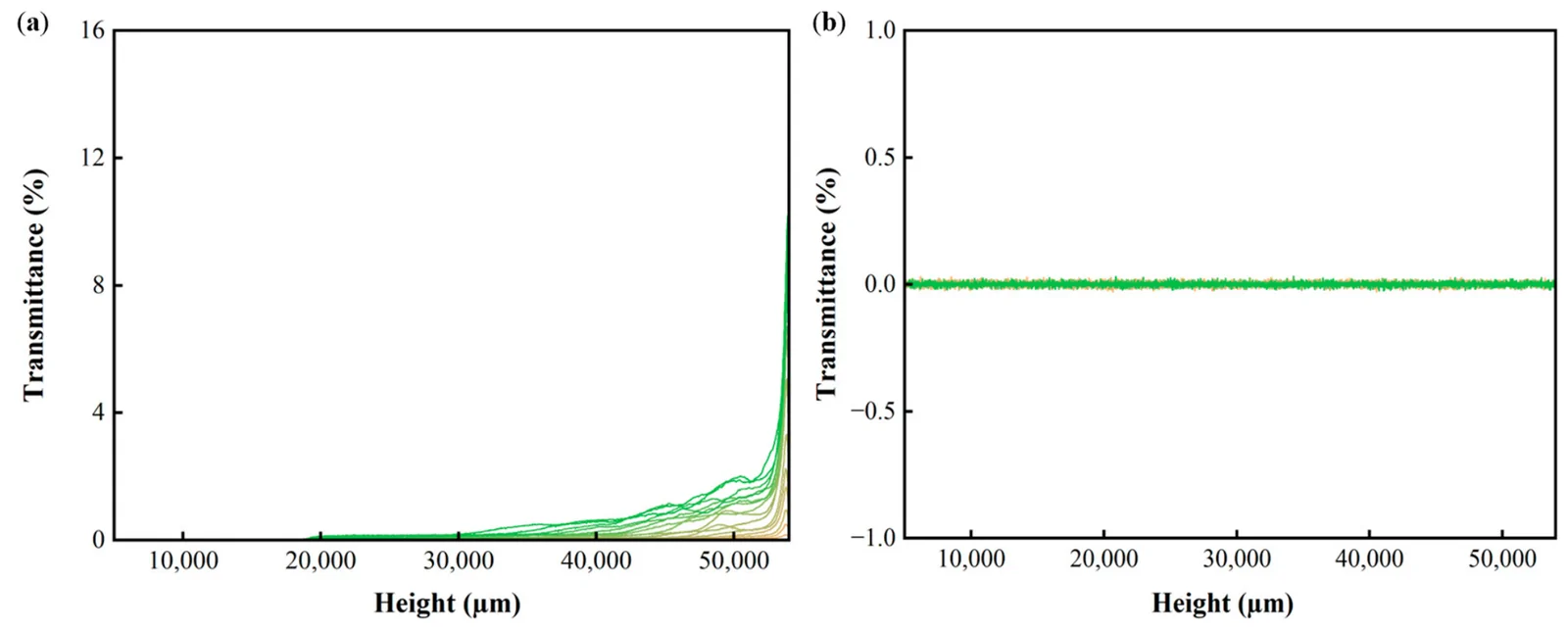
Multiple light scattering (MLS) transmittance profiles of aged T-CNF/barite (**a**) and MTCNF/barite (**b**) suspensions. The color gradient from yellow to green represents successive MLS scans recorded from 0 to 30 min at 1 min intervals. The differently colored lines correspond to transmittance profiles obtained at different measurement times, reflecting the temporal evolution of the suspension during the MLS measurement.

**Figure 7:**
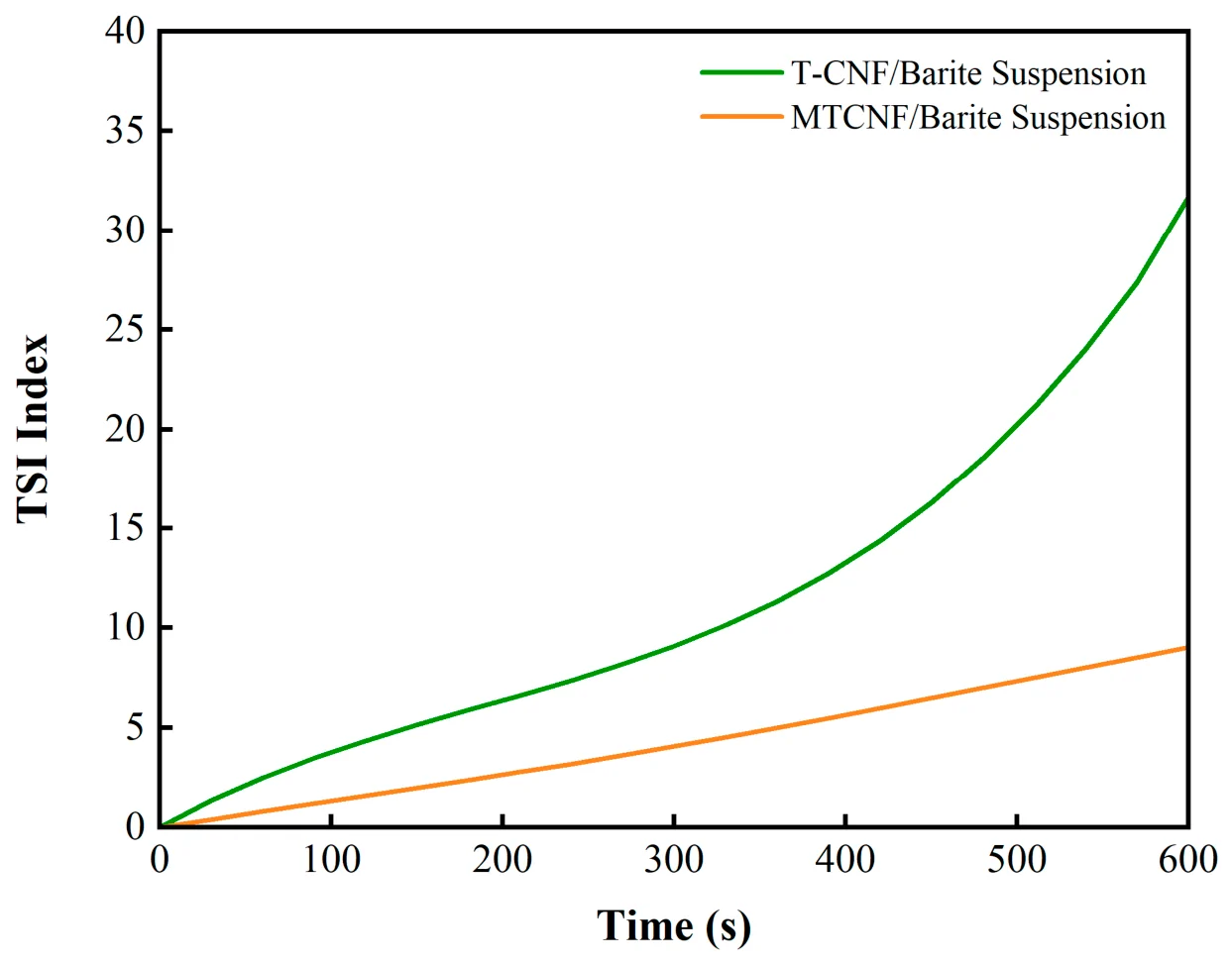
Turbiscan Stability Index (TSI) curves after thermal aging.

**Figure 8:**
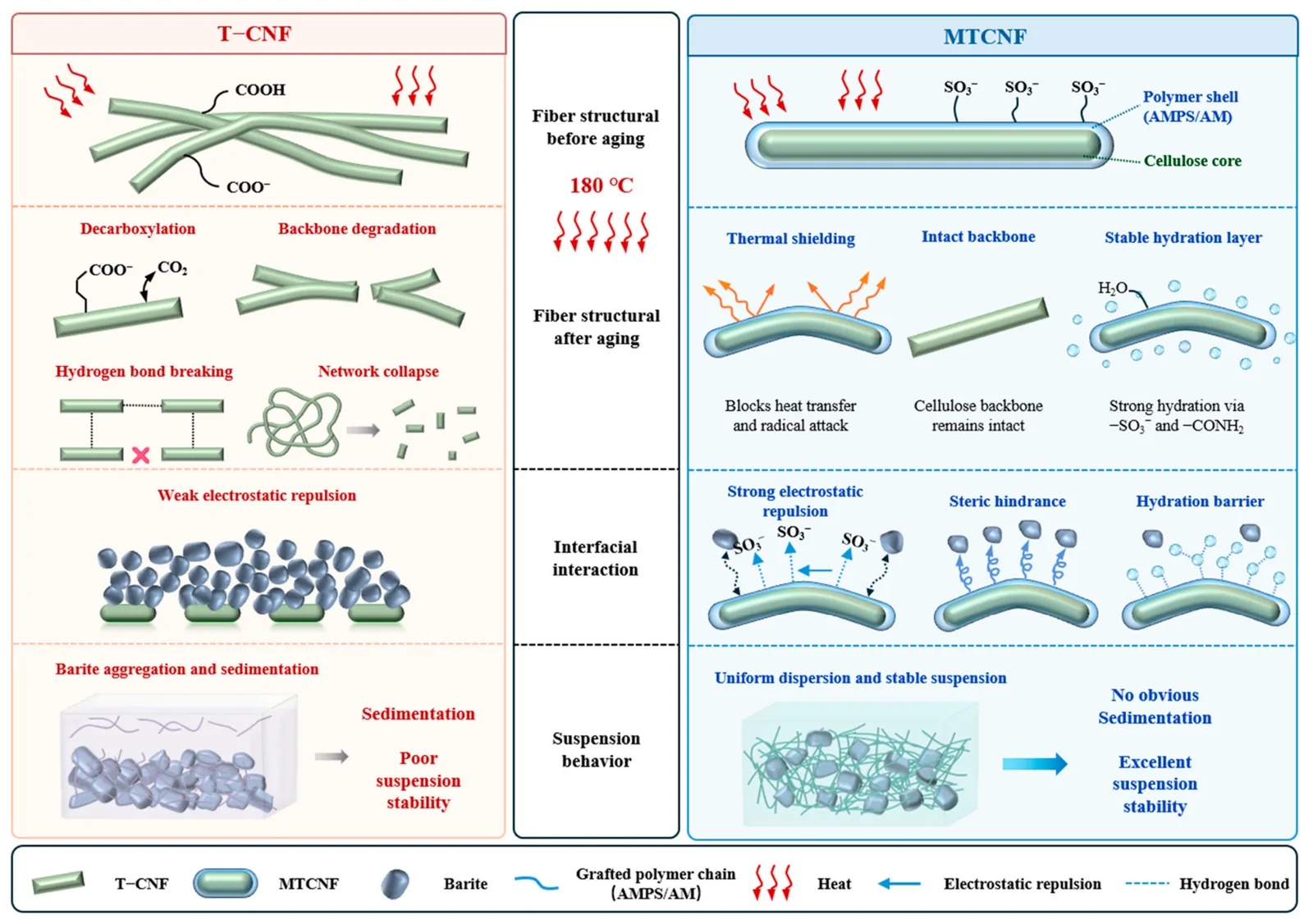
Proposed mechanism for the enhanced high-temperature suspension stabilization of barite by MTCNFs, illustrating the synergistic effects of enhanced electrostatic repulsion, hydrated steric stabilization, and a reinforced three-dimensional nanofiber network.

**Figure 9:**
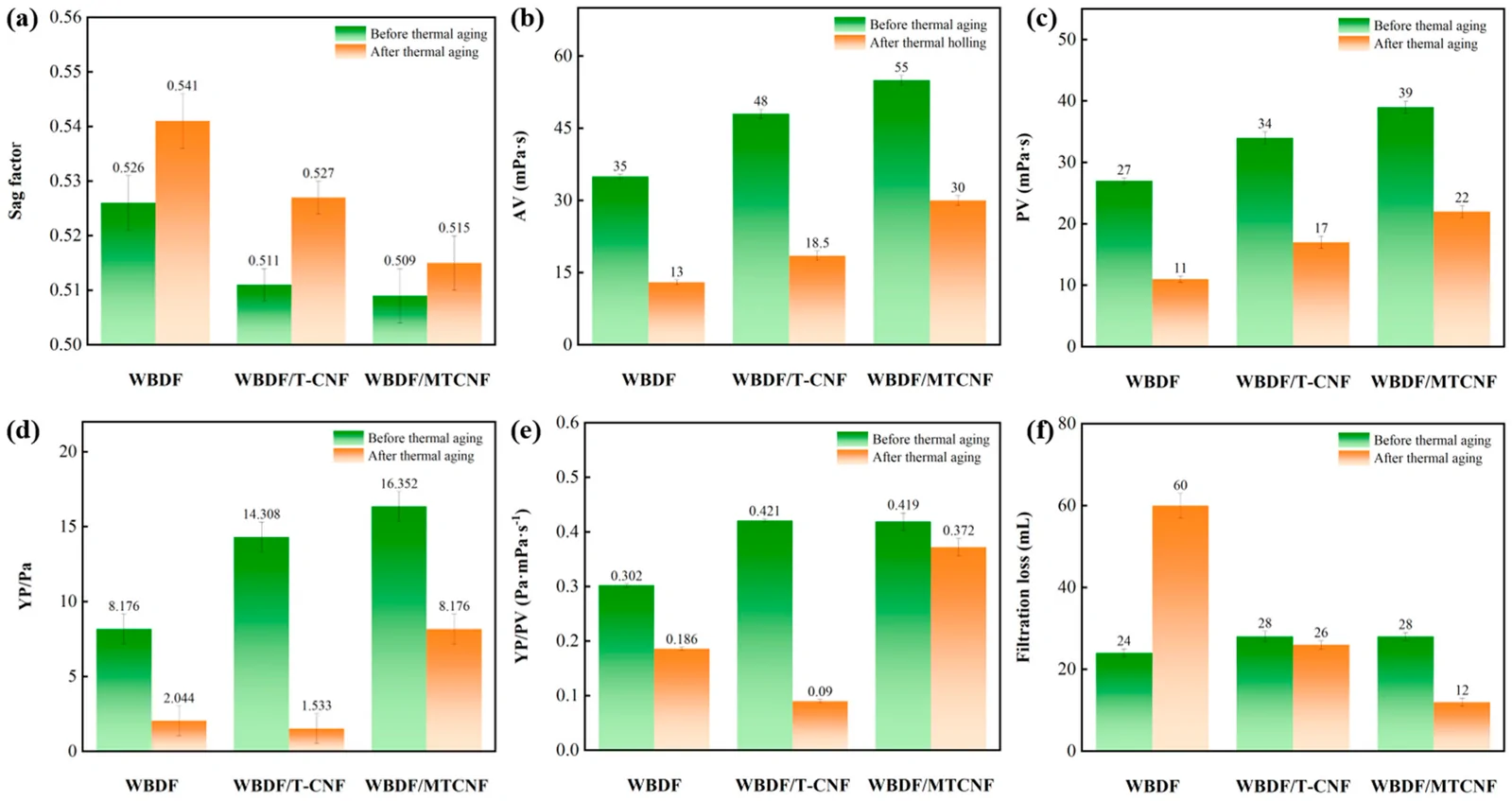
Performance of WBDFs containing TCNFs and MTCNFs before and after thermal aging: (**a**) sag factor, (**b**) apparent viscosity, (**c**) plastic viscosity, (**d**) yield point, (**e**) yield point/plastic viscosity ratio, and (**f**) filtration loss.

**Table 1:** Biodegradability of cellulose nanofibers and the corresponding WBDF systems.

Sample	CODcr (mg/L)	BOD_5_ (mg/L)	BOD_5_/CODcr/%	Degradability Assessment
T-CNFs	1.00 × 10^3^	4.75 × 10^2^	47.5	Easily degradable
MTCNFs	1.43 × 10^3^	6.45 × 10^2^	45.1	Easily degradable
WBDF/T-CNFs	2.83 × 10^3^	7.60 × 10^2^	26.9	Easily degradable
WBDF/MTCNF	3.21 × 10^3^	6.44 × 10^2^	20.1	Degradable
WBDF/HPAM	3.50 × 10^3^	5.25 × 10^2^	15.0	Relatively low degradable

## Data Availability

The original contributions presented in this study are included in the article. Further inquiries can be directed to the corresponding author.

## References

[1] Sun J., Yang J., Bai Y., Lyu K., Liu F. (2024). Research progress and development of deep and ultra-deep drilling fluid technology. Pet. Explor. Dev..

[2] Wang J., Wang R., Ren H., Ding Y., Liu J., Li Y., Yang Z., Shi H., Liao B., Li M.-C. (2026). Hollow silica nanospheres as wellbore thermal insulation materials in water-based drilling fluids. Colloids Surf. A Physicochem. Eng. Asp..

[3] Ali I., Khan M.A., Ding Y., Liu C., Li M.C. (2026). Recent Advances in Thermally Insulated Drilling Pipes: Materials, Design Strategies, and Future Directions. Polymers.

[4] Liu Z.-Y., Tian Y., Zhang X., Xiong B., Dai L., Wen J.-L., Yuan T.-Q. (2025). One-step synthesized filtration control agent for high-temperature and high-salt water-based drilling fluid. Sep. Purif. Technol..

[5] Liu F., Zhang Z., Liao B., Sun J., Khan M.A., Li M.-C. (2025). Recent advances in phase change microcapsules for oilfield applications. Adv. Geo-Energy Res..

[6] Martin C., Nourian A., Babaie M., Nasr G.G. (2024). Innovative drilling fluid containing sand grafted with a cationic surfactant capable of drilling high pressure and high temperature geothermal and petroleum wells. Geoenergy Sci. Eng..

[7] Li M., Ding Y., Sun J., Lv K., Huang X., Wang J., Zhang J., Yang L. (2025). Research progress and prospect of wellbore cooling technology. Acta Pet. Sin..

[8] Sun J., Xu G., Ding Y., Lyu K., Fan J., Li J. (2025). Advances and future prospects of shale oil and gas drilling fluid technology. Pet. Explor. Dev..

[9] Al Jaberi J., Bageri B., Elkatatny S., Patil S. (2023). Primary Investigation of Barite-Weighted Water-Based Drilling Fluid Properties. ACS Omega.

[10] Yang L.-L., Wu Y.-P., Liu Z.-Y., Ou Z.-T., Chen H.-Z., Jiang G.-C., Dong T.-F., Yu C.-J., Feng S.-J. (2025). Preparation, characterization and application of modified barite grafted with lipophilic polymers for oil-based drilling fluids. Geoenergy Sci. Eng..

[11] Guo J.H., Sang Y., Meng B.Q., Xia L.B., Wang Y.S., Ma C.Y., Tan T.Y., Yang B. (2025). Study on the Wellbore Instability Mechanism in the Longtan Formation with Soft/Hard Thin Interlayers in the South Sichuan Basin. Processes.

[12] Al Jaber J., Bageri B., Alsaleem A., Alshehri Y., Patil S. (2025). Insights Into Barite Particle Size and Perlite Integration to Advanced Drilling Fluids Efficiency. J. Energy Resour. Technol. Part B.

[13] Kwon Y.-M., Cho G.-C., Chang I. (2025). Effects of xanthan gum treatment on sedimentation and consolidation of kaolinite aggregates. J. Rock Mech. Geotech. Eng..

[14] An Y., Pang S., Zhao L., Li T., Yan L. (2025). Filtration loss mechanisms of polyacrylamide-based copolymers in high-temperature drilling fluid. J. Polym. Eng..

[15] Wang H., Li M., Qin X., Sun K., Zhang C. (2022). Hydrophobic association copolymer as viscosifier for high-temperature high-pressure anti-sedimentation performance of water-based drilling fluid. Polym. Eng. Sci..

[16] Noor A.A., Khan M.A., Zhang Y., Lv K., Sun J., Liu C., Li M.-C. (2025). Modified natural polymers as additives in high-temperature drilling fluids: A review. Int. J. Biol. Macromol..

[17] Lu G., Zhao J., Li S., Chen Y., Li C., Wang Y., Li D. (2021). Incorporation of Partially Hydrolyzed Polyacrylamide with Zwitterionic Units and Poly(Ethylene Glycol) Units Toward Enhanced Tolerances to High Salinity and High Temperature. Front. Mater..

[18] Antony Jose S., Cowan N., Davidson M., Godina G., Smith I., Xin J., Menezes P.L. (2025). A Comprehensive Review on Cellulose Nanofibers, Nanomaterials, and Composites: Manufacturing, Properties, and Applications. Nanomaterials.

[19] Ding Y., Liu C., Xu G., Xin M., Khan M.A., Wu B., Lv K., Sun J., Liu C., Yang L. (2026). Biomass-based porous materials for thermal insulation. Mater. Sci. Eng. R.

[20] Wen D., Zhai S., Xingyue L., Sijie L., Xu C., Zhang L., Wu W. (2025). Synthesis of CNC based one-dimensional SERS probes and evaluation of cell imaging performance. J. For. Eng..

[21] Shi Y., Jiang G.H. (2024). Effect of ZIF-8/nanocellulose on deodorization of bamboo-wood composite fibers. J. For. Eng..

[22] Zhang Y., Lv K., Sun J., Liu C., Li M.-C. (2025). Lignin-containing cellulose nanofibers as stabilizers in Pickering emulsion-based drilling fluids. Int. J. Biol. Macromol..

[23] Li M.-C., Yan H., Dai L., Yang D., Khan M.A., Liao B., Lv K., Sun J., Liu C. (2025). Cellulose Nanofibers and Xanthan Gum as Stabilizers in Pickering Foam Drilling Fluids for Enhanced Coalbed Methane Development. Langmuir.

[24] Liu C., Li M., Mei C., Chen W., Han J., Yue Y., Ren S., French A.D., Aita G.M., Eggleston G. (2020). Cellulose nanofibers from rapidly microwave-delignified energy cane bagasse and their application in drilling fluids as rheology and filtration modifiers. Ind. Crops Prod..

[25] Abbas A.K. (2021). Experimental investigation of cuttings transport with nanocomposite water-based drilling fluids modified by cellulose nanoparticles. Colloids Surf. A Physicochem. Eng. Asp..

[26] Li M.-C., Tang Z., Liu C., Huang R., Koo M.S., Zhou G., Wu Q. (2020). Water-Redispersible Cellulose Nanofiber and Polyanionic Cellulose Hybrids for High-Performance Water-Based Drilling Fluids. Ind. Eng. Chem. Res..

[27] Sun J., Wen Z., Khan M.A., Lv K., Shen H., Dai L., Li Y., Ding Y., Liu C., Li M.-C. (2024). A review of cellulose nanomaterial-stabilized Pickering foam: Formation, properties, and emerging oilfield applications. Int. J. Biol. Macromol..

[28] Khan M.A., Li M.-C., Lv K., Sun J., Liu C., Liu X., Shen H., Dai L., Lalji S.M. (2024). Cellulose derivatives as environmentally-friendly additives in water-based drilling fluids: A review. Carbohydr. Polym..

[29] Guo R., Li H., Tian X., Fan Y., Wang C., Huan S. (2024). Preparation of lignin/nanocellulose hybrid particles by co-precipitation method. J. For. Eng..

[30] Yang D., Khan M.A., Liao B., Shi X., Lv K., Sun J., Liu C., Li M.-C. (2025). Cellulose nanofibers as anti-sagging agents in water-based drilling fluids. Int. J. Biol. Macromol..

[31] Khan M.A., Lv K., Sun J., Liu F., Shen H., Ding Y., Noor A.A., Lalji S.M., Liu C., Li M.-C. (2025). Enhanced Filtration Performance of Cellulose Nanofiber/Bentonite Water-Based Drilling Fluids with Temperature- and Salt-Resistant Polymers for Extreme Conditions. SPE J..

[32] Dai L., Sun J., Lv K., Liu F., Bai Y., Liao B., Yang D., Liu C., Li M.-C. (2025). Cellulose nanofiber-reinforced supramolecular polymer gels for temporary plugging of fractured oil and gas reservoirs. Carbohydr. Polym..

[33] Liu X., Yuan Z., Wang A., Wang C., Qu J., Chen B., Wei B., Kapu N.S., Wen Y. (2020). Cellulose nanofibril-polymer hybrids for protecting drilling fluid at high salinity and high temperature. Carbohydr. Polym..

[34] American Petroleum Institute (API) (2009). Recommended Practice for Field Testing Water-Based Drilling Fluids, API RP 13B-1.

[35] Zhou J., Ma Y., Chen J., Cai Z., Qi L., Cui J., Deng S., Ouyang W., Fang Z., Qiu X. (2025). Supramolecular Scale Hydrophilicity Regulation Enabling Efficient Dewatering and Assembly of Nanocellulose into Dense and Strong Bulk Materials as Sustainable Plastic Substitutes. Adv. Mater..

[36] Shu F., Guo Y., Huang L., Zhou M., Zhang G., Yu H., Zhang J., Yang F. (2022). Production of lignin-containing nanocellulose from poplar using ternary deep eutectic solvents pretreatment. Ind. Crops Prod..

[37] Schwanninger M., Rodrigues J.C., Pereira H., Hinterstoisser B. (2004). Effects of short-time vibratory ball milling on the shape of FT-IR spectra of wood and cellulose. Vib. Spectrosc..

[38] Ma H., Wang W., Yu W., Kong F., Wang S., Liu K., Zhou Z., Wei G., Wang X., Liu Y. (2025). Preparation of dispersible TEMPO-CNF ultrafine powder and its application in achieving superhydrophobicity. Cellulose.

[39] Pourmahdi M., Abdollahi M., Nasiri A. (2023). Effect of lignin source and initiation conditions on graft copolymerization of lignin with acrylamide and performance of graft copolymer as additive in water- based drilling fluid. J. Pet. Sci. Eng..

[40] Liu P., Yang X., Chen W., Hao Y. (2024). Preparation of the modified chitosan flocculant introduced acryloyloxyethyl trimethyl ammonium chloride and 2-acrylamido-2-methyl propane sulfonic acid for the treatment of papermaking wastewater. Colloids Surf. A Physicochem. Eng. Asp..

[41] Park J., Lee D., Hwang K., Lee J., Lee T.-J., Kim Y., Kim J.H., Lee J., Youe W.-J., Chun S.-J. (2024). Effect of catalyst and oxidant concentrations in a TEMPO oxidation system on the production of cellulose nanofibers††Electronic supplementary information (ESI) available. RSC Adv..

[42] Zhang Y., Zhao Y., Zhao Z., Shan M., Xu B., Xue H., Xu J., Wu F., He Q. (2026). Characterization of Cellulose Nanofibrils Prepared by Direct TEMPO-Mediated Oxidation of Coffee Grounds. J. Polym. Mater..

[43] Zhang T., Hui L., Zhao Z., Zhu R., Xin J., Zhao M. (2026). TEMPO-oxidized cellulose nanofiber/chitosan composite films stabilized by cumin essential oil-loaded Pickering emulsions for multifunctional food preservation. Int. J. Biol. Macromol..

[44] Osolnik U., Vek V., Humar M., Oven P., Poljanšek I. (2025). Tailoring bionanocomposite film functionality using cellulose nanofibrils and bioactive wood extractives. Cellulose.

[45] Yu Y., Zhang H., Xu P., Zhang X., Wang H., Hu M., Guo J. (2025). Effect of Ultra-High Temperature Degradation on the Physical Properties and Chemical Structure of an AMPS-Based Copolymer Oil-Well Cement Additive PADIM in Aqueous Solution. Polymers.

[46] Manimarn M., Norizan M.N., Haafiz M.K., Adam M.R., Norrrahim F., Feizal V. (2024). Critical assessment of the thermal stability and degradation of chemically functionalized nanocellulose-based polymer nanocomposites. Nanotechnol. Rev..

[47] Wang B., Gong S., Shi C., Hao P., Cheng Y., Li B., Li D. (2025). Study on Preparation and Mechanism of High Temperature and High Salt Resistance Fluid Loss Reducer. ACS Omega.

[48] Ramírez Brenes R.G., Chaves L.d.S., Bojorge N., Pereira N. (2023). Endo-Exoglucanase Synergism for Cellulose Nanofibril Production Assessment and Characterization. Molecules.

[49] Xu J., You F.-C., Zhou S.-S. (2022). Study of a high-temperature and high-density water-based drilling fluid system based on non-sulfonated plant polymers. Polymers.

[50] Mohamed A., Al-Afnan S., Elkatatny S., Hussein I. (2020). Prevention of Barite Sag in Water-Based Drilling Fluids by A Urea-Based Additive for Drilling Deep Formations. Sustainability.

[51] Pinheiro J.A., Marques N.d.N., Villetti M.A., Balaban R.d.C. (2021). Polymer-Decorated Cellulose Nanocrystals as Environmentally Friendly Additives for Olefin-Based Drilling Fluids. Int. J. Mol. Sci..

[52] Wang X., Lei Q., Luo J., Wang P., Xiao P., Ye Y., Wu X., Liu Y., Zhang G. (2021). Application of Nanocellulose in Oilfield Chemistry. ACS Omega.

[53] Qasim U., Sirviö J.A., Suopajärvi T., Hu L., Pratiwi F.W., Lin M.K.T.H., Anghelescu-Hakala A., Ronkainen V.-P., Xu C., Liimatainen H. (2024). A multifunctional biogenic films and coatings from synergistic aqueous dispersion of wood-derived suberin and cellulose nanofibers. Carbohydr. Polym..

[54] Camargos C.H.M., Poggi G., Chelazzi D., Baglioni P., Rezende C.A. (2022). Strategies to mitigate the synergistic effects of moist-heat aging on TEMPO-oxidized nanocellulose. Polym. Degrad. Stab..

[55] Solhi L., Guccini V., Heise K., Solala I., Niinivaara E., Xu W., Mihhels K., Kro M., Meng Z., Wohlert J. (2023). Understanding Nanocellulose–Water Interactions: Turning a Detriment into an Asset. Chem. Rev..

[56] Arola S., Kou Z., Rooijakkers B.J.M., Velagapudi R., Sammalkorpi M., Linder M.B. (2022). On the mechanism for the highly sensitive response of cellulose nanofiber hydrogels to the presence of ionic solutes. Cellulose.

[57] Deng C., Du G., Li X., Xi J., Lin C. (2022). Effect of high temperature and high pressure on the biodegradability and biotoxicity of typical additives in drilling fluid. J. Pet. Sci. Eng..

[58] Abolore R.S., Jaiswal S., Jaiswal A.K. (2024). Green and sustainable pretreatment methods for cellulose extraction from lignocellulosic biomass and its applications: A review. Carbohydr. Polym. Technol. Appl..

[59] Al-Kindi S., Al-Bahry S., Al-Wahaibi Y., Taura U., Joshi S. (2022). Partially hydrolyzed polyacrylamide: Enhanced oil recovery applications, oil-field produced water pollution, and possible solutions. Environ. Monit. Assess..

[60] Song T., Li S., Yin Z., Bao M., Lu J., Li Y. (2021). Hydrolyzed polyacrylamide-containing wastewater treatment using ozone reactor-upflow anaerobic sludge blanket reactor-aerobic biofilm reactor multistage treatment system. Environ. Pollut..

